# Evaluation of Transfusion Pyrexia: A Review of Differential Diagnosis and Management

**DOI:** 10.5402/2012/524040

**Published:** 2012-10-15

**Authors:** Oladimeji P. Arewa

**Affiliations:** Department of Laboratory Medicine and Pathology, University of Alberta, Edmonton, AB, Canada T6G 2R3

## Abstract

*Background/purpose*. Transfusion pyrexia (fever) is an important clinical sign/symptom occurring either as an isolated event or as part of a constellation of signs and symptoms in relation to blood transfusion. It is an important cause of morbidity and may be an important sign of life-threatening complications of blood transfusion. Pyrexia is often a reason for the discontinuation of a blood transfusion episode, and adequate evaluation remains a challenge for clinicians. The decision to stop a blood transfusion episode on account of fever is often a difficult one. This paper reviews the differential diagnosis of transfusion pyrexia (TP), the pathogenesis as well as current management measures. *Study selection and data source*. Literature sources include medical texts, journals, dissertations, and internet-based electronic materials *Results and conclusion*. Adequate evaluation of pyrexia accompanying blood transfusion remains a challenge for clinicians. An algorithm to assist the clinician in the evaluation of fever occurring in a blood transfusion recipient is developed and presented. Continuous medical education is necessary for clinicians towards improved patient care in transfusion medicine.

## 1. Introduction 

Blood transfusion is an important life-saving measure in clinical practice. It is nonetheless sometimes complicated by adverse events. Pyrexia (fever) is an important clinical sign/symptom that occurs either as an isolated event or as part of a constellation of signs and symptoms of some hazards of blood transfusion. Transfusion pyrexia (TP) is the elevation of temperature ≥1°C from baseline or temperature >38°C, with or without chills or rigors occurring in a recipient of a unit of blood or blood component with no other explanation other than the transfused unit [1]. 

The correct evaluation of fever in a blood transfusion recipient is important as this sign/symptom is manifested by several distinct clinical entities varying from simple febrile non-haemolytic transfusions (FNHTR) to life threatening complications as transfusion related acute lung injury (TRALI) and acute haemolytic transfusion reactions [2]. In addition, transfusion pyrexia is an independent factor that predicts platelet recovery, increment, or survival in transfusion recipients [3, 4]. The decision to stop the administration of blood in a case of transfusion pyrexia is often a difficult one. Many, but not all cases, can be tolerated by the transfusion recipient with supportive care and analgesics [5, 6]. Unfortunately, reliable guidelines are not available to help with this decision [6]. The onset of pyrexia in a transfusion recipient correlates with the pathophysiology of the specific etiology; thus, while it could be a few minutes as a result of the presence of accumulated cytokines in the transfused unit (in the case of FNHTR), its onset could be delayed up to two weeks as a result of transfusion transmitted malaria and even up to 4 weeks for transfusion associated graft versus host disease (TAGVHD) in which engraftment of viable T cell is central to the pathogenesis of the entity. 

The appropriate evaluation of pyrexia after a reasonable interval from the transfusion event in particular requires a high index of suspicion by the clinician as the transfusion may inadvertently be completely overlooked in relation to the febrile episode. This paper highlights the important differential diagnoses and the approach to the management.

## 2. Differential Diagnoses of Transfusion Pyrexia

An important fundamental in the approach to the differential diagnoses and management in all cases is the early detection of fever arising from transfusion. An optimal approach to management should incorporate quarter hourly vital signs monitoring from the onset of transfusion commencement for the first 30 minutes and half hourly monitoring thereafter till transfusion is ended. Monitoring of vital signs chart after transfusion is equally important in the days immediately following the transfusion.

The following are important differential diagnoses of transfusion pyrexia.

- Febrile non Haemolytic transfusion reaction.
- Haemolyic transfusion reactions (Immediate and Delayed).
- Bacterial contamination (Bacteraemia).
- Transfusion transmitted malaria.
- Transfusion related acute lung injury (TRALI).
- Transfusion associated graft versus host disease (TAGVHD).

## 3. Febrile Nonhaemolytic Transfusion Reaction (FNHTR)

 The occurrence of a febrile nonhaemolytic reaction is an important complication of a blood component transfusion because of its possible confusion with other more dangerous transfusion reactions, such as acute haemolysis, sepsis, and transfusion-related acute lung injury (TRALI), with which it shares common features [2]. Febrile non-haemolytic reactions were thought to be mainly due to antileucocyte antibodies, with antibodies directed against HLA antigens, or against granulocyte-specific antigens [7–9]. As a result, universal leucoreduction of blood components has been advocated by some to reduce the incidence of febrile nonhaemolytic transfusion reaction [7]. Some other workers found no significant impact of leucodepletion of red cells on the incidence of FNHTR [10, 11].

Recent studies, however, have shown that the dominant factor determining the risk of a febrile reaction was not white cell contamination, but the age of the component which predisposes to accumulation of cytokines in the transfused unit [12–14]. Another frequent cause of a non-haemolytic febrile reaction is sensitization to white cell or platelet antigens [15]. A rise in temperature may be the sole symptom, but the recipient may suffer chills, rigors, or headache. These reactions are usually troublesome but not life-threatening. Febrile responses have also been reported as being more common in patients receiving platelet transfusions than red cell transfusion [14]. This has been attributed to raised levels of CD-154, a potent inducer of cyclooxygenase 2 (Cox-2) enzyme and thus PGE-2, an important fever inducer [16, 17]. Furthermore, the incidence of FNHTR with single-donor platelet (SDP) is much less as compared with random donor platelet (RDP), and transfusion of platelet concentrate as soon as possible after collection minimized the risk of accumulation of cytokines [17]. 

 The optimal strategy for dealing with FNHTR is controversial [18, 19]. Those who advocate halting the transfusion while screening tests are undertaken to exclude acute haemolysis, sepsis, and TRALI, with resumption of the transfusion of the same unit of blood product, risk not completing the transfusion, while those who advocate the routine permanent disconnection of the unit from the administration set, returning it to the blood bank and substituting a different unit to complete the patient's transfusion, risk exposing the patient to multiple donors thus increasing the recipient's risk of alloimmunization [20] and transfusion transmitted disease acquisition [21], as well as potentially compromising the inventory of the blood bank [2]. Both of these strategies, apart from the risks they pose, imply more discomfort for the patient and more cost for the patient and the health care provider. In a study of transfusion reactions at a tertiary hospital in Nigeria, 70% of discontinued transfusions were as a result of FNHTR; out of these, 58% of the discontinued transfusion episodes were successfully completed with tepid sponge and antipyretic cover following review by a haematologist [5]. The association of allergic reaction with a febrile episode is not uncommon [5, 22]. In such instances, the addition of antihistamine and or hydrocortisone is beneficial to the management of the patient. In some cases, the symptoms of an FNHTR may be sufficiently severe that the patient becomes apprehensive and reluctant to have further transfusions; therefore, elimination of FNHTRs will be beneficial to these patients. Acetaminophen, a common nonprescription nonsteroidal anti-inflammatory drug (NSAID) is sometimes given as a premedication and has been reported to lead to a significant reduction in the incidence of FNHTR. The issue of premedication with antipyretics for FNHTR has been a subject of debate amongst transfusionists. While some have posited that premedication can mask fever and thus make it difficult to quickly identify some more dangerous conditions such as TRALI, acute haemolysis, and sepsis, some other workers have found no evidence to corroborate such fears [2]. Furthermore, even if the thermal response to these reactions can be suppressed by antipyretics, other manifestations of these reactions remain, as hypotension, haemolysis, rigors, nausea, vomiting, and tachycardia are not suppressed by antipyretics [2]. Still, others advocate the use of pretransfusion medication, but only in those patients who have had prior febrile episodes [23]. To do so though means denying those patients a useful prophylaxis during their original transfusion. For the health care provider, antipyretic premedications also bring about some benefits. The lower rate of reaction makes feasible a policy of the return of implicated units to the blood bank for laboratory evaluation. Secondly, the use of antipyretic medications reduces the chance of symptoms of an FNHTR that may obscure the clinical findings of a patient's underlying illness and place additional burden on the hospital's resources, as well as the resources of medical, nursing, and laboratory personnel [2]. Furthermore, termination of a prescribed necessary transfusion, with resultant wasting of the products, will also be avoided. Finally, the use of intravenous pethidine could be indicated in some cases of troublesome febrile non-haemolytic reactions with severe rigors especially associated with platelet transfusions [24]. This has been found particularly useful in cancer patients who require large volumes of platelet concentrate transfusions while on myeloablative therapy or recovering from the transplant. However, a haemolytic transfusion event as well as bacterial contamination *must* be excluded before the use of pethidine for the management of febrile non-haemolytic transfusion reactions [24].

## 4. Haemolytic Transfusion Reactions

Haemolytic reactions could be *immediate *or *delayed*, depending on whether signs and symptoms occur within or after 24 hrs. Immediate haemolytic transfusion reactions usually result from ABO incompatibility. It is believed to be the most dangerous type of transfusion reaction and highly avoidable. They are usually due to clerical or administrative error [25]. The haemolytic antibodies are generally IgM or rarely complement binding IgG. *Pyrexia* is a prominent feature in the constellation of signs and symptoms. There is pain at the site of the intravenous access as well as severe constricting chest and loin pains, tachycardia, hypotension, and haemoglobinemia with subsequent haemoglobinuria and hyperbilirubinemia. Uncontrollable bleeding due to disseminated intravascular coagulation may occur and may actually be the only sign of a haemolytic transfusion reaction in an unconscious or anesthetized patient. 

The severity of the reaction is dependent on the site of red cell destruction, which is dependent on antibody characteristics. Intravascular red cell destruction associated with the activation of full-complement cascade is the most dangerous type of hemolytic reaction [25]; however, weak antibodies that do not seem to be clinically significant in vitro have been reported to cause severe acute hemolytic transfusion reactions [26]. Delayed hemolytic transfusion reactions (DHTRs) are characterized by a triad of pyrexia, anaemia, and hyperbilirubinemia and are well-recognized hazards of blood transfusion that may occur as a result of an anamnestic immune response [27–29]. DHTRs are seen more frequently in patients with sickle cell disorders (SCD) and haemoglobinopathies than in other groups of patients [30]. Such reactions are neither predictable nor preventable; usually an individual has been previously sensitized to one or more red cell antigens by transfusion or pregnancy. Antibody is not detectable in routine pre-transfusion screening, but the transfusion of blood, containing antigens to which the recipient has previously been sensitized provokes a brisk anamnestic response. However, Patten et al. [31] reported a case of DHTR resulting from a primary immune response. Awareness of DHTR in particular for the patient at risk can limit wastage of scarce resources by the patient and the medical, nursing, and laboratory personnel in “septic workup” [25]. Management of DHTR is mainly supportive, and no definitive treatment may be necessary. 

The management of immediate haemolytic transfusion reaction is an emergency. A transfusion reaction form should be completed, and notification of the blood bank at the time the reaction is suspected is mandatory to allow prompt investigation. Adequate attention must be given to the urinary output of the patient with strict input-output monitoring. Such patients may benefit from intensive care unit (ICU) management. Diuretics and positive inotropic drugs such as dobutamine and adrenaline are invaluable. Where the facility is available, haemodialysis is helpful as circulating immune complexes which are generated as a result of the haemolytic reaction are removed in the process thus attenuating the inflammatory response. Immediate haemolytic transfusion reactions could be prevented through the avoidance of clerical errors by the laboratory staff as well as the clinical staff before the administration of blood.

## 5. Bacterial Contamination

Transfusion pyrexia could be a sign or symptom of the systemic inflammatory response syndrome (SIRS) complicating the transfusion episode as a result of bacterial contamination of blood for transfusion (septicaemia) [32, 33]. Transfusion of heavily contaminated blood will usually lead to high fever, collapse, shock, and hemorrhagic phenomena due to disseminated intravascular coagulation (DIC). A number of gram-negative, psychrophilic, and endotoxin-producing contaminants found readily in dirt and soil (pseudomonades, coliforms) may very rarely enter a unit and grow readily under the storage conditions of blood and even more rapidly at room temperature. Severe fulminant toxic symptoms can be seen after transfusion of blood contaminated by *Staphylococcus* or *Yersinia*. *Yersinia enterocolitica *grows well in red cell components due to its dependence on citrate and iron [21].

Bacterial contamination is commoner with platelet transfusion apparently due to the storage temperature for platelet concentrates (22°C–24°C), a temperature conducive for rapid proliferation of most bacteria contaminants often arising from inadequate cleaning of the phlebotomy site on the donor. Prevention of bacterial contamination of blood component is the most important aspect of management. The use of single donor platelets as against the preparation of platelet concentrates from pooled donor (random donor platelets) has been shown to reduce the risk of bacterial contamination from donor skin flora or asymptomatic bacteraemia [34]. Careful examination of the blood bag before transfusion could lead to identification of a contaminated blood bag as a result of colour change of the donor unit, and such units should not be transfused. Inactivation of pathogen in platelet concentrate using photochemical techniques is targeted not only to bacteria but also to a wide spectrum of viruses, spirochetes, parasites, and leukocytes. 

Pathogen inactivation is a proactive method which anticipates the contamination of the blood pool by emerging pathogens [35]. In cases where transfusion of a contaminated blood component has been inadvertently carried out, stopping the transfusion immediately reduces the bacteria load of the patient, and the hospital blood bank should be immediately notified. Completion of a transfusion report form is an important aspect of the management. After initial supportive care, blood cultures should be taken and broad-spectrum antimicrobials commenced. Laboratory investigation will include culture of the blood pack. Diagnosis is established by Gram stain and blood culture of both the blood component and the recipient. Further antibiotic administration should be guided by culture and sensitivity report.

## 6. Transfusion Transmitted Malaria

Malaria is one of several blood borne infections transmitted through blood transfusion. It is caused by *Plasmodium *spp. of which the most important is *Plasmodium falciparum*. The first case of transfusion transmitted malaria was reported in 1911 [36]. Transmission of this parasite through blood is important as only a small number of infected cells from the donor can lead to malaria in the recipient of the unit [37]. Transfusion-acquired *Plasmodium falciparum*-induced malaria fevers predispose to significant morbidity, not only after whole blood transfusion, but also after infusion of components, such as platelet cryoprecipitate and leucocytes, with the average incubation period being 7–10 days [38, 39]. This could, however, be up to three weeks in some cases. The risk of acquiring malaria via the transfusion of blood components is extremely low in nonendemic countries such as Canada and the United States. This is largely due to the strict donor deferral criteria. A transfusion malaria risk of 0.25 cases/million donor units has been estimated in the United States [39], with a fairly steady incidence of one to three cases per year reported by the United States Centers for Disease Control and Prevention (CDC) [40]. In contrast, the risk in endemic regions which include Central and South America, Hispaniola sub-Saharan Africa, the Indian subcontinent, the Middle East, Southeast Asia, and Oceania may be more than 50 cases/million donor units [32]. The actual prevalence of transfusion transmitted malaria in Nigeria is not known. However, the malaria endemic status of Nigeria makes the issue of donor deferral on account of malaria status unrealistic as exclusion would include nearly all eligible donors. Deferral policies for malaria are not practical for endemic areas [37, 41]. The symptoms developed by the recipient include *fever*, chills, headache, muscle aches, and malaise. A thick blood film is necessary to confirm the diagnosis. If the film is positive for malaria parasite, appropriate antimalaria therapy should be immediately instituted in accordance with the current treatment guidelines for the region.

## 7. Transfusion-Related Acute Lung Injury

In recent years, transfusion-related acute lung injury (TRALI) has developed from an almost unknown transfusion reaction to one of the most common cause of transfusion-related major morbidities and fatalities [42, 43]. TRALI, a condition also known as noncardiogenic pulmonary edema presents with *fever*, cough, tachypnea, tachycardia, wheeze, cyanosis, hypotension and evidence of pulmonary infiltrate on chest X-ray. A clinical definition of TRALI was established in 2004, based on acute respiratory distress, non-cardiogenic pulmonary edema temporal association with transfusion and hypoxaemia [42]. It could be confused as a case of severe anaphylaxis, and a high index of suspicion is needed to make the diagnosis. The onset typically occurs within 6 hours of transfusion, but most cases present within 1 to 2 hours. Transfusions of all blood products have been associated with the disease. 

The incidence of TRALI has been reported as 0.02% of all units or 0.16 of all patients, although it is believed to be underdiagnosed [44–46]. Clinical predisposing factors may be associated with the development of TRALI, as it has been observed more frequently in patients with sepsis, cancer, or patients who had received multiple transfusions [2]. Yang et al. [46] reported two cases of TRALI resulting from designated blood transfusion between mother and child and suggests that designated blood transfusion between multiparous mothers and children might add an additional transfusion-related risk owing to the higher likelihood of the HLA antibody-antigen specificity between mother and child. The pathophysiology is unclear but has been attributed to HLA antibodies, granulocyte antibodies, and more recently to biologically active mediators in stored blood components. Immune complexes are formed, and entering the pulmonary vascular bed stimulates the release of vasoactive substances that cause the leakage of fluid into alveolar spaces, activation of complement, leukostasis, and activation of polymorphonuclear neutrophils [42, 43]. Diagnosis is confirmed with antibodies found in donor plasma against panel of normal granulocytes [44]. Management is generally supportive and similar to that for adult respiratory distress syndrome. Ventilatory and hemodynamic assistance are utilized as required, and with good ventilatory support, most of the symptoms resolve within 96 hrs of instituting such assistance. Although steroids are often given as part of treatment in TRALI, there are no clear indications for the use of corticosteroids, and their use remains controversial in this setting [42]. Additional blood component therapy should not be withheld if clear indications for transfusion exist. There is, however, enough evidence to warrant permanent deferral of a donor whose donated unit is frequently implicated in the etiology of TRALI [45].

## 8. Transfusion-Associated Graft versus Host Disease

 Ta-GVHD occurs when donor lymphocytes in cellular blood products engraft in a susceptible transfusion recipient. Thus, an index of suspicion is necessary when blood components are given to at risk categories of patients (see Table 1). The clinical syndrome comprises fever, skin rash, pancytopenia, abnormal liver function, and diarrhoea. Transfusion-associated GVHD occurs when viable T lymphocytes in blood components are transfused, and they engraft and react against the recipient's tissues causing damage to target organs especially bone marrow, skin, liver, and gastrointestinal tract, and the recipient is unable to reject the donor lymphocytes. Normally, recipient lymphocytes are capable of recognising foreign HLA and prevent the development of a donor antihost immune response. Two factors may allow such a response to develop. Firstly, sharing of HLA-haplotypes between donor and recipient which occurs when HLA-selected components are transfused or when donations are obtained from relatives. This is particularly true when HLA homozygous blood components are transfused [47, 48]. The second factor is defective recipient cell-mediated immunity which may be inherited, for example, severe combined immune deficiency-SCID or acquired, for example, Hodgkin's disease [49]. Other factors which may be relevant are the age of the component as the number of viable lymphocytes diminishes with storage. Lymphocyte dose is important, but leucodepletion does not prevent TA-GVHD [49]. Not all cases of acquired immune suppression states are, however, at risk for TaGVHD, thus there is no need for irradiation of components for transfusion in such cases (Table 2). Generally, however, the most commonly reported setting for Ta-GVHD is in immunocompetent recipients of blood from biologically related (directed) or HLA identical donors. The most frequent reports of TA-GVHD in immunocompetent individuals are from Japan, where there is a greater HLA homogeneity in the general population [48]. Transfusion-associated GVHD carries a very poor prognosis; it is fatal in over 90% of cases [47]. Gamma irradiation of cellular blood components is the recommended method of preventing this complication [50].

 The blood bank must be appraised of the immune status, or diagnosis, of the patient so that cellular components intended for transfusion of immunocompromised patients and blood components from designated donors will be irradiated. The dose of gamma irradiation should be a minimum of 25 Gy to any part of the blood component container [51]. Irradiation of blood red cell containing components, however, decreases the red cell survival and increases the potassium of the component. There is no apparent effect on platelet survival. fresh frozen plasma [FFP] and cryoprecipitated AHG (CRYO) need not be irradiated, because these components do not contain enough viable lymphocytes to cause GVHD [52].

**Figure 1:**
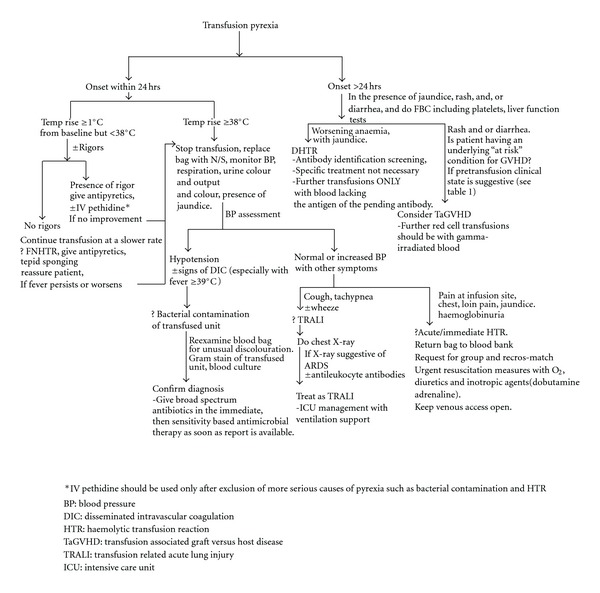
Algorithm for Evaluation of Transfusion Pyrexia.

In conclusion, transfusion pyrexia is an important sign and or symptom of blood transfusion that should be properly evaluated by the transfusionist. A good understanding of the pathophysiology of the differential diagnoses is indispensable to the correct evaluation of fever in blood transfusion. The algorithm developed in (Figure 1), may be used in patient evaluation in order to institute appropriate management. Continuous medical education in transfusion medicine is necessary for improved patient care.

## Figures and Tables

**Table 1:** Immunosuppressive conditions with relative risk for TaGVHD.

At risk groups of patients	
(1) Autologous bone marrow/stem cell transplant recipients	
(2) Allogeneic bone marrow/stem cell transplantrecipients	
(3) Hodgkin's disease	
(4) B-cell malignancies (non-Hodgkin's lymphoma, multiple myeloma, Waldenstrom's macroglobulinemia, ALL)	
(5) Fludarabine, cladribine therapy	
(6) Directed donations from blood relatives	
(7) HLA matched platelets	
(8) Congenital immunodeficiency disorders (*SCID, Wiskott-Aldrich*)	
(9) Intrauterine transfusions	
(10) Granulocyte transfusions in infants	

**Table 2:** Immunosuppresive states with no risk for TaGVHD.

No indication for component irradiation
(1) AIDS/HIV infection
(2) Full term neonates
(3) Acute leukaemia without transplantation
(4) Aplastic anaemia
